# Free choice of healthcare providers in the Netherlands is both a goal in itself and a precondition: modelling the policy assumptions underlying the promotion of patient choice through documentary analysis and interviews

**DOI:** 10.1186/1472-6963-12-441

**Published:** 2012-12-03

**Authors:** Aafke Victoor, Roland D Friele, Diana MJ Delnoij, Jany JDJM Rademakers

**Affiliations:** 1 NIVEL, Netherlands Institute for Health Services Research, P.O. Box 1568, 3500 BN, Utrecht, Netherlands; 2 Tilburg School of Social and Behavioural Sciences, Tranzo, Tilburg University, P.O. Box 90153, 5000 LE, Tilburg, Netherlands; 3 Centre for Consumer Experience in Health Care (CKZ), P.O. Box 1568, 3500 BN, Utrecht, Netherlands

**Keywords:** Choice behavior, Patients, Patient satisfaction, Quality of healthcare, Healthcare reform, Netherlands

## Abstract

**Background:**

In the Netherlands in 2006, a health insurance system reform took place in which regulated competition between insurers and providers is key. In this context, the government placed greater emphasis on patients being able to choose health insurers and providers as a precondition for competition. Patient choice became an instrument instead of solely a goal in itself. In the current study, we investigated the concept of ‘patient choice’ of healthcare providers, as postulated in the supporting documentation for this reform, because we wanted to try to understand the assumptions policy makers had regarding patient choice of healthcare providers.

**Methods:**

We searched policy documents for assumptions made by policy makers about patient choice of healthcare providers that underlie the health insurance system reform. Additionally, we held interviews with people who were involved in or closely followed the reform.

**Results:**

Our study shows that the government paid much more attention to the instrumental goal of patient choice. Patients are assumed to be able to choose a provider rationally if a number of conditions are satisfied, e.g. the availability of enough comparative information. To help ensure those conditions were met, the Dutch government and other parties implemented a variety of supporting instruments.

**Conclusions:**

Various instruments have been put in place to ensure that patients can act as consumers on the healthcare market. Much less attention has been paid to the willingness and ability of patients to choose, i.e. choice as a value. There was also relatively little attention paid to the consequences on equity of outcomes if some patient groups are less inclined or able to choose actively.

## Background

In most northwest European countries, such as the Netherlands, Scandinavia and the UK, actively choosing a healthcare provider was traditionally not common. In the Netherlands for instance, although patients have always had free choice of doctor, in practice general practitioners long had fixed patient lists (linked to capitation payments) and visits to a medical specialist were (and still are) only possible after referral by a GP. However, initiatives have been taken recently in all these countries to extend patients’ ability to choose their provider, to encourage them to make an active choice and to support them in the process of making their choice [1-7].

There are two main reasons why patient choice is promoted [8]. Firstly, choice of provider gained importance as something that patients value. Because today’s patients are more demanding, they want a more active role in their own healthcare [9-11]. National governments have responded to this development with legislation for patient rights that strengthens their role or, in other words, empowers them. Giving patients the right and possibility to choose is one aspect of patient empowerment [8,9,11]. It gives patients a strong instrument to influence their healthcare [11,12].

Secondly, engaging patients in their own healthcare is also seen as the best way to ensure sustainability of health systems, to promote quality improvement and to shorten waiting times [2,8,11]. This is the instrumental use of patient choice. Patients were expected to ‘vote with their feet’ [13] by choosing only those healthcare providers that offer the best care, based on the comparative information available on quality and costs. This selection prompts providers to compete for patients by improving the care they deliver, because, when the care they deliver is not optimal, patients may ‘punish’ them by going elsewhere (exit) [8,12,14-16].

In the Netherlands, encouraging patient choice also has multiple goals [8]. In the 1970s, the Dutch government set itself the aims of explicitly developing policy on patients and legislation for patients’ rights as part of the emancipatory developments in large parts of Europe and the USA to empower various groups within society, e.g. women, homosexuals, and also patients. This political tendency meant that choice of provider gained importance as something patients valued [8,17]. During the late 1980s, the instrumental use of patient choice gained importance. This occurred as part of a government plan to reform the Dutch health insurance system into a system in which regulated competition between healthcare providers and insurers is key: it was assumed to be a way to guarantee the efficiency, quality and accessibility of healthcare [2,8,18,19]. All these developments together resulted in plans for a healthcare system in which patient choice is important, both in its own right and as a precondition for competition between providers.

**Figure 1:**
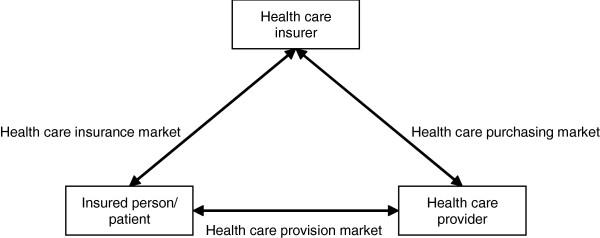
The healthcare market.

Regulated competition was implemented in the Netherlands in 2006 and resembles the ‘managed competition’ model described by Enthoven [20]. The change was mainly supported by two acts: the Health Insurance Act (Zvw) and the Act on Market Regulation in Healthcare (Wmg). Before the change, patients, healthcare providers and insurers were in a triangular relationship and the Dutch government regulated the supply and costs of healthcare and the relationships between the three parties. People were insured through two very different health insurance schemes, i.e. a social health insurance scheme (ZFW) and an alternative private health insurance scheme (PHI) (Table 1) [21,22]. The changes introduced three interdependent markets in healthcare in which the three different parties (i.e. providers, insurers and consumers) were assigned new roles (Figure 1) [23]. The first market is the healthcare provision market, where well-informed patients were assigned the responsibility to ‘vote with their feet’ by selecting the healthcare providers they preferred [23-25]. The second market is the healthcare purchasing market, where healthcare providers offering a high value-for-money ratio are contracted by insurers. The third market is the healthcare insurance market. In this market, well-informed consumers have to choose between health insurers and insurance products [19,23]. Only a single health insurance scheme exists for the whole population, but people can make several choices, e.g. between benefits in kind and benefits in cash (Table 1). The principle of ‘voting with your feet’ also applies in this market. Patients are assumed to choose selectively between health insurers based on e.g. the range of providers contracted and the insurers’ quality of service. This is assumed to encourage insurers to compete for consumers by contracting care providers that offer good value for money. Instead of regulating the supply of healthcare, the government creates a level playing field in which market forces can play a role [19].

**Table 1:** Key elements of the health insurance schemes in the old insurance system compared with the new insurance system

	**Old system**		**New system**
	Social health insurance (ZFW)	Alternative private health insurance (PHI)	Private social health insurance
Insured people	People under a certain income ceiling (two-thirds of the population)	People above a certain income ceiling (one-third of the population)	The whole population
Mandatory/voluntary	Mandatory primary healthcare package and voluntary additional healthcare package	Voluntary	Mandatory primary healthcare package and voluntary additional healthcare package
Premium rating	Income-dependent (85%) and community rating (15%)	Dependent on the risk profile of the person requesting the insurance	Income-dependent (50%) and community rating (50%)
Benefits in kind/ benefits in cash	Benefits in kind	Benefits in cash	Insurers are allowed to offer both
Voluntary policy excess	No	Yes	Yes
Insurer choice	Those insured could change insurer and additional insurance product yearly, but in practice choice options were limited	Those insured could change insurer and insurance product yearly	Those insured can change insurer and additional insurance product yearly
Provider choice	Free choice among contracted providers	Free choice among all providers, but may receive only partial reimbursement	Free choice among all providers, but may receive only partial reimbursement

### Research focus

It is interesting to investigate whether promoting patient choice of healthcare providers has had its desired effects. The first step is to make explicit what the underlying assumptions are about how patient choice is meant to work and what impact it is expected to have. These assumptions need to be understood first, because they determine the indicators needed to evaluate the effectiveness of the policy. In the current study, we will model the assumptions underlying the health insurance system changes, focusing on the role of patients’ choice of providers. In this process, we will answer the following questions: 

▪ What did policy makers aim to accomplish by promoting patient choice of providers?

▪ What determinants were assumed to influence patient choice of providers?

▪ How did policy makers assume more patient choice could be promoted?

▪ What possible side-effects of the promotion of patient choice were discussed?

Although there are several scientific papers that describe the assumptions underlying the promotion of patient choice in various countries, e.g. [8,14-16,26], as far as we know, only a few aimed to model policy assumptions by analysing policy documents in combination with interviews with key figures, e.g. [26]. Even fewer tried to model patient choice in the Netherlands using this method. The current paper therefore expands the body of literature about public policy evaluation, adds to existing knowledge about regulated competition in healthcare, and will enable future research on the validity of this policy.

## Method

### Modelling the policy assumptions

Various methods are described in the literature for modelling the assumptions underlying public policy and how they are interrelated [27,28]. In general, these methods assume that such a model consists of the following three parts, which correspond to the research questions: 

▪ The problem/goals: which problems does the policy aim to solve?

▪ Causal assumptions in the form of if-then-propositions: if a certain condition is true or a certain component of the policy is implemented, then the following consequence is assumed.

▪ Final assumptions in the form of if-then-propositions: if a certain goal is to be accomplished, then this step has to be taken.

### Data collection

In order to model the assumptions of the key policy makers and their interrelations, we followed the ‘policy-scientific approach’ described by Leeuw [28]. Additionally, we modelled the possible side-effects of the promotion of patient choice that were discussed. The method described by Leeuw [28] relies on an analysis of policy documents and interviews with key policy makers. In accordance with this approach, expressions from key policy makers about the concept ‘patient choice’ were first extracted from a variety of documents (Appendix A). All the documents consulted are direct products of the ministry of Health and were published between 2004 and 2007, because the Wmg and Zvw were developed during this period. In all, 62 documents were consulted. Program texts of the instruments mentioned in the documents analysed were consulted to gain a deeper understanding of the goals of these instruments. The sources that we used for the analysis and that we refer to in the text are shown in the References [29-86].

**Figure 2:**
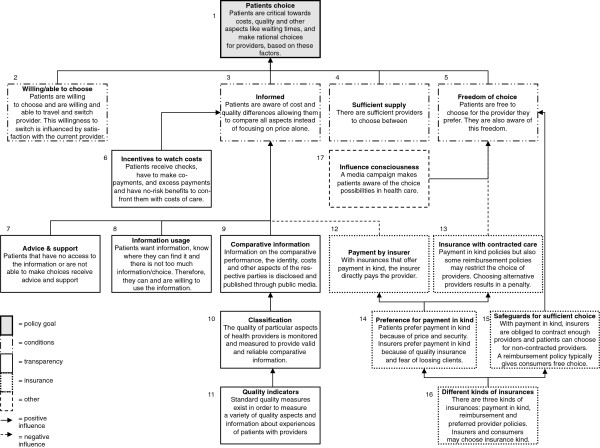
Reconstruction of the causal propositions.

In addition to the analysis of the policy documents, the model that resulted from this analysis was shown to seven people who were either involved in the development of the current health insurance system or whose professional position enabled them to follow this development closely (Appendix A). All were asked whether our model (i.e. Figure 2 and Table 2) was plausible and whether we had missed out any assumptions.

**Table 2:** Reconstruction of the final propositions

**Legal instruments**	**Condition**	**Communicative instruments**	**Condition**	**Financial instruments**	**Condition**
Insurers may choose the policy forms they offer [45]	F	Media campaign from the government about the new health insurance system [37,42-44]	F	Financial incentives (e.g. co-payments) [37,39,40,43,49,56,63]	I
Patients have the right to choose from the available policy forms [45]	F	Developing quality/performance indicators [30,31,41,43,44,57,58,70,71]	I	Subsidies from government [44,72-75]	I
With a payment-in-kind policy, providers are obliged to deliver care as agreed within a reasonable time and at a reasonable distance [76]	F	Developing comparative information, e.g. for kiesbeter.nl or the Healthcare Inspectorate (IGZ) [31,49,57,77-79]	I		
With a payment-in-kind policy or a preferred provider policy, patients have the right to receive compensation when they choose a non-contracted provider in the Netherlands and in Europe [50]	F	Comparative information, e.g. kiesbeter.nl, physical desks, telephone, papers, healthcare providers, insurers and user organizations (peer contact and information provision) [30,37,38,41-43,46,53,54,57,59,70,80,81]	I		
With a reimbursement policy or a personal budget, patients are allowed to choose their preferred provider freely without intervention from the insurer [42]	F	Advice and support for people who are unable to choose independently (e.g. by MEE) [56,74,82]	I		
Providers are obliged to publish comparative information [53]	I				
Providers are obliged to make the information understandable, effective and correct [54]	I				
The information providers provide may not be misleading and must comply with the legislation [55]	I				

*W* = willing/able to choose; *F* = freedom of choice; *I* = Informed.

### Analysis

The problems, goals, causal and final assumptions and side-effects were extracted from the assembled expressions. All assumptions were reformulated in the form of propositions and these propositions were used to construct a model. Assumptions and instruments that overlapped were combined and side issues were excluded. All the authors together discussed the resulting model. Disagreements were discussed until consensus was reached.

The interviews were transcribed and sent to the interviewees for correction and approval. Where necessary, adjustments were made to our model with the information from the approved documents.

### Ethical considerations

Our research complied with the Helsinki Declaration where applicable. According to the Dutch ‘Medical Research involving human subjects Act’, our study did not require ethical approval from a medical ethics committee [87]. Verbal informed consent was obtained from all interviewees, who were informed of their right to withdraw from the study at any time they wished without penalty. All the interviews were voice recorded with the permission of the interviewees, and the resulting recordings and transcripts were kept confidential. Additionally, we asked the interviewees to correct and approve the transcripts so that we could use them for our study. Only those transcripts that were approved were used in our study. We removed all personal identification information in this paper.

## Results

### The political and policy problems/goals

The Dutch government reformed the health insurance system and promoted patient choice. If patient choice is part of the solution, what then were the problems in the ‘old’ Dutch health insurance system? The explanatory memorandums accompanying the two laws that supported the system change – the Zvw [29] and the Wmg [30] – mention a variety of problems in the ‘old’ Dutch health insurance system.

Promoting patient choice was expected to solve two important political problems. Firstly, the Dutch healthcare system was centralised or state-oriented, i.e. the Dutch government regulated the supply of healthcare instead of entrusting it to the patients and healthcare insurers and providers. Consequently, there were no opportunities or reasons for the three different parties to affect each other’s practices and results. Healthcare was therefore unresponsive to patients, innovation and flexibility were curtailed, there was no incentive for providers to improve their quality and efficiency, etcetera. Secondly, the asymmetrical, paternalistic patient-doctor relationship urged patients to leave decisions about their care to their doctors.

The first problem above was expected to be solved by the introduction of ‘regulated competition’ in healthcare, which was intended to replace the governmental regulation of supply. Patient choice was promoted as one element of regulated competition. The promotion of patient choice can therefore be considered as an instrument to achieve the goal of ‘regulated competition in healthcare’. Regulated competition, in turn, was considered to be an instrument for achieving higher-level goals, e.g. more efficient (including cost control) and more accessible healthcare of higher quality. The fact that patient choice was also promoted to solve the second problem shows that it was considered as a goal in itself as well, i.e. it was assumed to strengthen the autonomy of patients. The interviewees also stressed the fact that the concept of ‘patient choice’ is both an instrument to improve the quality, efficiency and accessibility of care on a macro level and a goal in its own right, in other words a goal in itself. Enabling choice of providers makes it easier for patients to match care to their preferences [33-36].

Although patient choice of providers was part of the solution to the political problems, several policy problems were encountered while trying to promote it, e.g.: 

▪ patients do not have enough choice options;

▪ patients do not have the freedom to make choices between providers and insurers;

▪ patients are insufficiently aware of their rights;

▪ there is no standardised method to measure the quality of the healthcare providers and providers may fail to provide this information;

▪ there is no level playing field in which insurers, providers and patients can play their roles.

To solve the policy problems and, in the end, to encourage patient choice and introduce regulated competition, the Zvw and - as the cornerstone of regulated competition - the Wmg were developed [30-32].

### The causal propositions

How can patient choice help to introduce competition and, in the end, achieve the higher-level goals (e.g. more efficient and more accessible healthcare of higher quality)? There are a number of assumptions about patient choice as a mechanism for quality improvement and cost containment. For instance, the government assumes that patients are willing to fit their care to their needs and are critical about certain characteristics of healthcare providers, such as the costs, quality and waiting times. Based on information about these characteristics, patients choose a provider rationally. When dissatisfied, they ‘vote with their feet’ by switching to a provider that fits their preferences better. This behaviour tells providers about patients’ levels of satisfaction with them and (because they want to obtain or keep patients as their clients) prompts them to match the care on offer to the wishes of patients [29-31,37-41].

Figure 2 shows the causal propositions that are part of the model and how they are interrelated. The interviews did not result in major changes, since the model was deemed plausible. However, the interviewees did not agree with each other on whether patients were assumed to pay attention to the costs of healthcare providers (Box 3) and whether patients’ cost awareness was heightened partly in order to make them focus on the costs of providers when choosing between them (Box 6). We will discuss this apparent ambiguity below.

To briefly summarise Figure 2, the policy goal we are concerned about is patient choice (Box 1). Various conditions are assumed to lead to patient choice (Boxes 2–5). A range of factors and instruments influence whether the conditions are satisfied (Boxes 6–16). Some have to do with the transparency of healthcare and lead to well-informed patients (Boxes 6–11). Others have to do with the healthcare insurer and/or insurance (Boxes 12 to 16). The majority of these factors influence the degree to which patients are free to choose a healthcare provider. The last box (17) represents the media campaign set up by government to make people aware of the possibilities of choice in healthcare [37,42-44].

### The final propositions

The Dutch government made various final assumptions. Patient choice is a goal in itself, but is also needed to introduce competition and to help achieve other public goals, e.g. ‘improving and safeguarding the quality, efficiency and accessibility of healthcare’ and ‘controlling the development of costs in healthcare’ [30,32]. In order to enable patients to choose a provider critically and actively, the four conditions should be satisfied (Boxes 2 to 5 in Figure 2). Figure 2 and Table 2 show the instruments that needed to be implemented in order to fulfil the conditions [29]. We divided the instruments into legal, communicative and financial instruments, which is a widely recognised division in the literature [27]. We did not investigate the ‘sufficient supply’ condition any further in this paper, because we intended to focus on instruments directed at the patient, but it is assumed to be an essential condition for choice and the government implemented several instruments to satisfy this condition [29]. It should also be noted that while the government deemed patient choice a goal in itself, they mainly focused on patient choice within the context of regulated competition. Although patient choice already was a goal in itself, all the instruments for patient choice were only implemented when patient choice became a part of regulated competition. Maybe because policy makers focused mainly on patient choice as an instrument, they did not implement any instruments to satisfy the condition of being ‘willing and able to choose’. They assumed that patients were indeed willing to make active choices in order to receive high-quality care [37]. Again, we checked the plausibility of our model with the interviewees. This did not result in any major changes.

Almost all the instruments affecting the ‘freedom of choice’ condition have to do with the healthcare insurer and/or insurance. Insurers are free to offer any policy form and patients are free to choose between the available policy forms [45]. The most important difference in policy forms is between those based on direct payment (payment in kind) and those based on restitution. The former guarantee the insured person access to healthcare providers from a list of providers contracted by the health insurer. The latter guarantee the insured person reimbursement of costs incurred. In practice, this implies a free choice of provider, but those insured may receive only partial reimbursement of the healthcare costs they incur [23-25]. Patients’ freedom of choice is thus determined by the type of policy they have [29,37,40-42,46-48]. Even so, under the payment-in-kind scheme, the insured persons are legally allowed to choose non-contracted providers (but they may potentially receive only partial reimbursement of costs) and, in the case of a payment-in-kind policy, insurers have to contract enough providers because they are obliged to deliver care within a reasonable time and at a reasonable distance [29,37,40-43,46,48-52]. A media campaign was set up by government to make the public aware of the possibilities of choice in healthcare [37,42-44].

The instruments affecting whether patients are informed all have to do with ‘transparency’, which refers to the availability of comparative information about the costs and performance in terms of effectiveness, safety and patients’ experiences with healthcare providers [23], [30,31]. Providers are obliged to publish understandable, effective and correct comparative information about quality and costs that is not misleading and does not undermine health legislation [30,38,41,46,53-55]. Because patients have different information preferences and a number of parties have developed various quality or performance indicators and comparative information, a plethora of information for patients has been produced [31,35]. Patients can consult the information, e.g. on websites of user organisations, providers and insurers and in newspapers. Additionally, to provide a single general portal that patients can consult, the government financed the creation of the well-promoted website ‘kiesbeter.nl’ (‘choose better’). People without Internet access were assumed to request information by phone or at physical desks or to request help from healthcare providers, insurers and user organisations [30,31,37-39,41-44,46,48,56-62]. Besides making comparative information available, people’s cost awareness was assumed to be heightened and they were made partly responsible for the costs they incur through financial incentives, e.g. they receive cheques, have to make co-payments and pay policy excesses and have no-risk benefits. This way, their critical attitude towards the costs of healthcare would be influenced positively and, consequently, they would be motivated to demand care only when they really need it [29,37,39,40,43,49,63,64].

### Side-effects of the policy

Several side-effects of the policy on the promotion of patient choice of healthcare providers are mentioned in the policy documents. For instance, policy makers assumed that not every patient has Internet access or is able to search the Internet, assess the various alternative providers and make an informed decision. This may lead to inequalities in the accessibility of the comparative information as well as in patients’ ability to choose. Because many patients eventually will not choose, the competitive pressure will be diminished [37,65,66]. All these side-effects are listed in Table 3.

**Table 3:** The side-effects of the policy regarding the enhancement of patient choice of healthcare providers that are mentioned in the policy documents

**Condition**	**Risk**	**Effect**
Willingness/ability to travel/choose	Some patients are not willing or able to travel or choose.	There may not be enough competitive pressure [41,51].
	There is an urgent situation.	Patients do not have time to search providers [56].
Sufficient choice	Healthcare providers and insurers enlarge (especially high quality-providers) or merge. Additionally, the plethora of rules implemented to regulate the market will lead to diminished entrepreneurial activity.	Patients do not have sufficient choice options [41,83] and costs will increase [84,85]. Consequently, there may not be enough competitive pressure [41,51].
	Patients have too many choice options.	Patients may delay choice [84].
Transparency – quality	The comparative information that is to be developed will be opaque, excessive, incomprehensible, not comparable, scattered and the various healthcare providers often are disparate.	Patients are unable to assess the quality of the providers and consequently cannot be critical about quality, are unwilling to pay for quality and focus on price information instead [41,65,86]. Consequently, there will not be enough competitive pressure [38].
	Not every patient has Internet access or is able to search the Internet, assess the different options and make an informed decision.	Inequalities exist in the accessibility of the comparative information and ability to choose. Consequently, many patients will not choose and the competitive pressure will be diminished [37,65,66].
	Healthcare providers have to deliver a large amount of data.	Transparency is hindered [86].
	The bureaucracy of the system leads to possibilities for data to be manipulated.	Providers show strategic behaviour and commit fraud [83].
	Patients choose based solely on information about quality.	The relationship of mutual trust between patient and doctor is being undermined [83].
Transparency - costs	Patients do not get to see their healthcare costs, only have to pay a small premium.	Patients are often unaware of the costs that they incurred, which limits their cost awareness. This might diminish the influence of the financial incentives to avoid excessive care use [40,83,84].
Freedom of choice	Insurers contract a limited number of providers.	Patients will not have freedom of choice [36,40,41].
	Only the ‘rich’ are able to choose a policy which offers them free choice.	Inequalities exist in the freedom of choice patients have [40,85].
	Insurers do not buy high-cost care in order to fend off high-risk insured parties.	Inequalities exist in the choices people have [37].

### Ambiguous aspects

Although the interviewees perceived our model as plausible, they did not agree on issues concerning the context of our model, e.g. the definition of patient choice and its relative importance in the new health insurance system. We therefore reached the conclusion that some aspects of the policy are ambiguous. Firstly, patient choice as postulated in the policy documents refers to individual patients matching their care to their needs by actively choosing providers [37]. However, according to some interviewees, the concept of ‘patient choice’ refers to the indirect or collective influence of patients on providers as well: they merely make healthcare providers aware of the fact that patients are not dependent on them anymore, even though not all patients eventually choose [34,67]. However, in both views, patients who (may) change provider in order to improve the care they receive are expected to improve healthcare at the macro level [34,68].

Concerning the use of patient choice as an instrument or precondition, the policy documents were unclear about whether patients were expected to take costs into account when choosing a healthcare provider. The interviewees also did not agree on this matter. We kept costs in our model, but with the idea that (with payment-in-kind policies and preferred provider policies) patients may be expected only to be aware of the costs of healthcare (to prevent excessive care use) and to place the responsibility of keeping an eye on the costs of the individual providers on the insurer.

Finally, the ‘freedom of choice’ condition is ambiguous. When insurers only contract a limited number of healthcare providers, patients’ preferred alternatives may not be available anymore. Therefore, in our reconstruction, payment in kind policies or preferred provider policies have a negative influence on the freedom of choice. However, there is no consensus about how the government defines freedom of choice, i.e. as the availability of the preferred alternative or the quality of the available alternatives and clarity about this quality [33-35,68]. In our model, we adopted the first meaning, because it is often stated that a reimbursement policy increases the freedom of choice [36]. Although insurers might be better able to negotiate with insurers than individual patients, the freedom of choice of healthcare providers is assumed to be important for patients.

## Discussion

Patient choice of healthcare providers is an important theme, not only in the Netherlands but in the UK and Scandinavia as well [1-7]. To be able to evaluate whether promoting patient choice of healthcare providers has its desired effects, it must be clear exactly which effects are desired. For that reason, we modelled the assumptions underlying the promotion of patient choice of healthcare providers by analysing policy documents and interviewing key figures. We focused our analysis on the Netherlands. However, because much the same assumptions are made by policy makers in the other northern European countries as well [14,15,26], our analysis is also interesting for policy makers and researchers in those countries.

In the current paper, we answered four research questions. The first research question concerned the reasons for promoting patient choice. Patient choice of healthcare providers is one important element in a much broader system in which regulated competition between providers and insurers is key to controlling the development of costs and improving and safeguarding the quality, efficiency and accessibility of healthcare [31,32]. Within the context of regulated competition, patients are expected to behave as rational actors. This line of reasoning originates from the classical economic theory [20,68]. In addition, patient choice was deemed a ‘value’ or a goal in itself. However, in practice, the Dutch government did not really concern itself with this latter goal [37,88]. Because it was assumed that patients value choice, no instruments were implemented to encourage patients to choose. Even so, literature indicates that a number of patient groups are in reality less inclined or able to choose actively, which may affect the equity of outcomes from patient choice policies [8,12].

The second research question concerned the determinants that were assumed to influence patient choice. It was assumed that satisfying several conditions leads patients to choose a provider rationally. Those conditions are that patients are willing to choose and willing and able to travel and switch provider, that patients are informed, that there are sufficient healthcare providers to choose from and that patients are free to choose their healthcare provider. Regarding the third research question, i.e. how policy makers were to promote patient choice, the Dutch government and other parties implemented a variety of instruments to satisfy the conditions, thus creating a level playing field in which market forces could come into play. This resulted in a health insurance system that relies heavily on laws to regulate the market [68]. In our analysis, we did not include the supervisors of the healthcare market such as the Dutch Health Care Authority (NZa), because we wanted to focus on instruments directed at the patient. These supervisory bodies were, however, considered essential for markets to develop.

Concerning the fourth research question about the side-effects of the policy, several possible side-effects are documented in the policy documents. If these side-effects exist, diminished competitive pressure and a healthcare provision market that is not really working without governmental intervention may result. It is however striking that no discussion was documented about the role of equality, neither as a possible negative side-effect of patient choice nor as part of the argument for patient choice. In the UK, for instance, fairness/equality was part of the case made for patient choice. In several other countries, such as the Nordic countries, there was some concern about the likelihood that introducing choice would result in adjustment of the healthcare system in favour of certain patient groups (e.g. healthy, more highly educated, young people). Other types of patients would be ignored by the providers [8]. The fact that Dutch policy makers had no concerns about equity is especially interesting because they did expect differences in choice behaviour between different patient groups [31].

Because policy making is not a straightforward process, some aspects of the policy are ambiguous [89]. These ambiguities can have a variety of causes. Secondly, policy on the health insurance system change was not strictly defined; instead, some choices were left open [68]. One example is that the minister of VWS was unwilling to make a choice between insurance policy types and was ready to let ‘all the players on the market’ decide on the matter [36]. Thirdly, in policy documents, assumptions are made and words are used for concepts that cannot be grasped merely by reading written material about the subject [34]. For example, patient choice is a concept that refers to the indirect influence patients (the demand side) have on healthcare providers, but it is never explicitly defined as such. Fourthly, there might not be one single way to understand the policy; instead, words and assumptions that are used in it might have different meanings for different people. For some policy makers, patient choice refers to individual patients actively choosing a healthcare provider, while for others the concept refers to the threat of competitors that patients might choose. Finally, the development of the policy on health insurance system change has been a political process during which compromises had to be negotiated, for example regarding which goal of patient choice is the main focus. There are also other countries, in which patient choice has multiple goals, such as Scandinavia and the UK [2,8,10,11]. However, the Netherlands is unique, since patient choice as a goal in its own right conflicts with letting insurers contract providers in selectively. Whereas the latter is essential for the functioning of the new health insurance system and regulated competition [18], the former was also included in the policy as a goal in its own right [69].

### Healthcare provision and the insurance market

Although the current study focuses on the choice of providers, the healthcare insurance and provision markets are interrelated. However, the policy makers involved in the development of the current health insurance system tried to make sure that patients will always have a free choice of provider, independently of their insurance products (there may be some financial consequences). This makes it valid to analyse the healthcare provision market separately from the healthcare insurance market in the Dutch situation.

### Limitations, strengths and follow-up research

One limitation of this study is that we confined our analysis mainly to policy documents about the Wmg and the Zvw. This meant that we did not incorporate the history of the health insurance system changes. We partially solved this issue by consulting additional literature in order to put our reconstruction into context. Furthermore, we did not have the opportunity to interview the person who was the Minister of Health during the years that the health insurance system acquired its final form. A strong point of this research is, however, that we held interviews both with key figures involved in the health insurance system change and with people who followed this development closely.

Another strength of this paper is that, as far as we know, few scientific papers have been written either in the Netherlands or abroad that aimed to model the policy assumptions underlying the promotion of patient choice by combining policy document analysis with interviews with key figures. The current paper therefore expands the body of literature about public policy evaluation, adds to the existing knowledge about regulated competition in healthcare, and will enable future research on the validity of this policy, e.g. whether patients are indeed willing to choose their provider.

## Conclusion

Patient choice of healthcare providers is both a goal in its own right and a fundamental element in a system in which regulated competition between providers is key. Several instruments have been put in place to ensure that patients can act as consumers on the healthcare market: making sure that they are well-informed and that the insurance system poses no barriers. There has been much less attention for the willingness and ability of patients to choose, i.e. choice as a ‘value’. Also, the consequences on equity of outcomes if several patient groups are less inclined or capable to choose actively received little attention.

## Appendix A. Data collection

### Document search

The database ‘overheid.nl’ was searched for policy documents from the Dutch House of Representatives and the Dutch Senate concerning the Zvw, Wmg and policy on patients/consumers. This database contains all policy documents, policy letters and minutes of meetings of the Dutch House of Representatives and the Dutch Senate. The three searches consisted of the search strings ‘zorgverzekeringswet’, ‘marktordening gezondheidszorg’ and ‘patiënten/consumentenbeleid’. The searches were restricted to meeting minutes and official papers from the years 2004 to 2006, because the Wmg and Zvw were developed during this period. The 344 documents we initially found were reviewed on title to determine whether they concerned patient choice for healthcare providers. 58 documents met this criterion. Additionally, we analysed the political agendas of the Dutch Ministry of Health from the years 2004, 2005 and 2006 and a document from the Dutch Health Care Authority (NZa).

### Interviews

Seven people occupying the following functions during the years in which the system acquired its final form were interviewed: two policy makers working for the Ministry of Health involved in the reform of the Dutch healthcare system, a professor of the policy and organisation of mental healthcare in the Netherlands working for the institute of Health Policy & Management (iBMG) who advised the Dutch government about the new healthcare system, a professor of healthcare policy analysis at Maastricht University; the president of the Federation of Patients and Consumer Organisations in the Netherlands (NPCF), the president of the association of Dutch health insurers (Zorgverzekeraars Nederland (ZN)) and the chairman of the Council for Public Health and Health Care (RVZ).

## Competing interests

The authors declare that they have no competing interests.

## Authors’ contributions

AV participated in the design of the study, carried out the policy document search and selection process, conducted the interviews, analysed the documents, modelled the assumptions and drafted the manuscript. JR also participated in the design of the study and the modelling of the assumptions and helped to draft the manuscript. All authors participated in modelling the assumptions, drafting the manuscript and reading and approving the final manuscript.

## Pre-publication history

The pre-publication history for this paper can be accessed here:

http://www.biomedcentral.com/1472-6963/12/441/prepub
